# Role of β3-adrenergic receptors in the action of a tumour lipid mobilizing factor

**DOI:** 10.1038/sj.bjc.6600086

**Published:** 2002-02-01

**Authors:** S T Russell, K Hirai, M J Tisdale

**Affiliations:** Pharmaceutical Sciences Research Institute, Aston University, Birmingham, B4 7ET, UK; Department of Obstetrics and Gynaecology, Osaka City University Medical School, Osaka 545-8585, Japan

**Keywords:** cachexia, lipid mobilizing factor, β3-adrenoceptor, energy metabolism

## Abstract

Induction of lipolysis in murine white adipocytes, and stimulation of adenylate cyclase in adipocyte plasma membranes, by a tumour-produced lipid mobilizing factor, was attenuated by low concentrations (10^−7^–10^−5^ M) of the specific β3-adrenoceptor antagonist SR59230A. Lipid mobilizing factor (250 nM) produced comparable increases in intracellular cyclic AMP in CHOK1 cells transfected with the human β3-adrenoceptor to that obtained with isoprenaline (1 nM). In both cases cyclic AMP production was attenuated by SR59230A confirming that the effect is mediated through a β3-adrenoceptor. A non-linear regression analysis of binding of lipid mobilizing factor to the β3-adrenoceptor showed a high affinity binding site with a Kd value 78±45 nM and a B_max_ value (282±1 fmole mg protein^−1^) comparable with that of other β3-adrenoceptor agonists. These results suggest that lipid mobilizing factor induces lipolysis through binding to a β3-adrenoceptor.

*British Journal of Cancer* (2002) **86**, 424–428. DOI: 10.1038/sj/bjc/6600086 www.bjcancer.com

© 2002 The Cancer Research Campaign

## 

Patients with cancer cachexia experience a dramatic loss of body fat as the condition progresses. A study of the body composition of lung cancer patients, who had lost 30% of their pre-illness stable weight, showed an 85% decrease in total body fat (Fearon, 1992), reflecting a prolonged catabolic state. Cancer patients with weight loss have been found to have an elevated level of a lipid mobilizing factor (LMF) in both serum and urine, which appears to parallel the weight loss (Groundwater *et al*, 1990). We have isolated LMF from the urine of cancer patients by a combination of ion exchange, exclusion and hydrophobic interaction chromatographies, and shown it to be homologous with the plasma protein Zn-α2-glycoprotein (ZAG) in primary sequence, electrophoretic mobility and immunoreactivity (Todorov *et al*, 1998). *In vivo* studies confirmed the ability of LMF to cause selective loss of carcass fat with no change in body water, and a tendency to increase the nonfat mass. LMF was characterized by the ability to stimulate lipolysis directly in isolated adipocytes, as a result of stimulation of adenylate cyclase in a GTP-dependent process (Hirai *et al*, 1998). The receptor for this interaction has not been characterized, but indirect evidence suggests that it may be a β3-adrenergic receptor (β3-AR).

Thus treatment of ob/ob mice with LMF, not only produced a specific depletion of the adipose mass together with an elevation of serum glycerol levels, but also an increased oxygen uptake by interscapular brown adipose tissue (BAT) (Hirai *et al*, 1998). Pharmacological studies indicate that the β-receptor responsible for the stimulation of oxygen consumption in BAT is exclusively of the β3-subtype (Howe, 1993). Induction of lipolysis in epididymal adipocytes by LMF was attenuated by the β-adrenergic receptor blocker propranolol (Khan and Tisdale, 1999), while the biphasic effect of GTP on cyclic AMP production by LMF in adipocyte plasma membranes suggests a receptor associated with both Gs and Gi. Only β3 and not β1-AR interact with Gi in adipocyte membranes (Granneman, 1995).

In the present study the ability of LMF to interact with the β3-AR has been studied both in white adipocytes and in CHO cells transfected with the human β3-AR.

## MATERIALS AND METHODS

### Patients

Urine was collected over a 24 h period from patients with unresectable pancreatic cancer and with a weight loss between 0.5 and 3 kg month^−1^. No patient had received radiotherapy or chemotherapy. Urine samples were stored at −20°C in the absence of preservatives prior to use.

### Chemicals

[α-^32^P]-ATP (sp. act. 20 Cimmol^−1^) and Na [^125^I] (sp. act. >15 Cimg^−1^ iodide) were purchased from Amersham Pharmacia Biotech (Bucks, UK). SR59230A was kindly donated by Dr L Manara of the Research Centre Sanofi Midy, Sanofi Winthrop S.p.A., Milan, Italy.

### Purification of LMF

LMF was purified from human urine using a combination of batch extraction on DEAE-cellulose and hydrophobic interaction chromatography (Todorov *et al*, 1998). Urine was centrifuged at 3000 *g* for 10 min to remove particulate material and was then diluted with 4 vol 10 mM Tris HCl, pH 8.0. DEAE-cellulose, previously activated by washing in 100 mM Tris HCl, pH 8.0 for 5 min was added to the diluted urine (10 *g* l^−1^ of original urine) and the mixture was stirred for 2 h at 4°C. The DEAE-cellulose was recovered by sedimentation by low speed centrifugation, and the LMF was eluted with 0.5 M NaCl in 10 mM Tris HCl, pH 8.0. The eluate was equilibrated and concentrated to 1 ml by ultrafiltration, in an Amicon filtration cell (Millipore (UK) Ltd, Watford, Herts, UK) containing a membrane filter with a molecular weight cut-off of 10 kDa, against PBS. Further purification was achieved using a Resource-Iso HPLC column (Pharmacia Biotech, St Albans, Herts, UK), employing a decreasing (NH_4_)_2_SO_4_ concentration from 1.5 M. Active fractions containing LMF eluted at 0.6 M (NH_4_)_2_SO_4_, and were desalted before use by washing five times against PBS using an Amicon filtration cell.

### Lipolytic assay

A single cell suspension of white adipocytes was prepared from the epididymal adipose tissue of ex-breeder male NMRI mice using collagenase digestion (Beck and Tisdale, 1987). Lipolytic activity was determined by measuring glycerol release (Wieland, 1974) after incubation of LMF with 10^5^−2×10^5^ adipocytes for 2 h at 37°C in 1 ml Krebs-Ringer bicarbonate buffer, pH 7.2. Control samples containing adipocytes alone were analyzed to determine the spontaneous glycerol release. Lipid mobilizing activity was expressed as μmole glycerol released 10^5^ adipocytes^−1^ 2 h^−1^.

### Adenylate cyclase assay

Plasma membranes were isolated from epididymal adipocytes, as previously described (Khan and Tisdale, 1999). Briefly isolated adipocytes were homogenized in 250 mM sucrose, 2 mM EGTA and 10 mM Tris HCl pH 7.4, followed by centrifugation at 30 000 **g** for 1 h at 4°C. The membrane pellet formed was isolated and separated from other organelle membranes on a self forming Percoll gradient, and the mixture was centrifuged at 10 000 **g** for 30 min at 4°C. The washed plasma membranes were diluted in 10 mM Tris HCl, pH 7.4, containing 250 mM sucrose, 2 mM EGTA and 4 μM phenylmethylsulphonylfluoride at 1–2 mg ml^−1^, and if not used immediately, snap frozen in liquid nitrogen and stored at −70°C until use. The adenylate cyclase assay was based on that developed by Salomon *et al* (1973) as previously described (Hirai *et al*, 1998). Briefly LMF was incubated for 10 min at 30°C together with plasma membrane in 25 mM Tris HCl, pH 7.5, 5 mM MgCl_2_, 10 μM GTP, 8 mM creatine phosphate, 16 units ml^−1^ creatine phosphokinase, 1 mM 3-isobutyl-1-methylxanthine and 1 mM [α-^32^P]-ATP (sp.act. 20 Cimmole^−1^) in a total volume of 100 μl. The reaction was terminated by the addition of 2% SDS, 40 mM ATP and 1.4 mM cyclic AMP. The cyclic AMP was isolated from the mixture using a combination of Dowex 50W8-400 and Alumina WN-3 columns, and the radioactivity was determined using a Tri-carb 2000A scintillation counter.

### Cyclic AMP determination

CHOK1 cells transfected with the human β3-AR, under the control of hygromycin, together with the b-gal reporter construct, selected for resistance to G418, were a gift from Dr Ian Waddell, Astra Zeneca, Macclesfield, Cheshire, UK. They were grown in Dulbecco's modified Eagles medium (DMEM) supplemented with 2 mM glutamine, 50 mg ml^−1^ hygromycin B and 200 mg ml^−1^ G418, under an atmosphere of 10% CO_2_ in air. For cyclic AMP assays cells were grown in 24 multi-well plates in 1 ml DMEM. Agonists were added to the wells and incubated for 30 min, after which the medium was removed and 0.5 ml 20 mM HEPES, pH 7.5, 5 mM EDTA and 0.1 mM isobutylmethylxanthine was added to each well. The plate was placed in a boiling water bath for 5 min and cooled on ice for 10 min. To 50 μl of the cell extract was added 2 μCi of [8-^3^H]-cyclic AMP (Amersham, UK) and 20 μg of cyclic AMP-dependent protein kinase (Sigma Chemical Co. Ltd, Dorset, UK) and incubated for 2 h at 4°C. Unbound cyclic AMP was removed by adsorption onto charcoal and the concentration of cyclic AMP in the sample determined by comparison with standard curves using known concentrations of cyclic AMP.

### Iodination of LMF with [^125^I]

One iodo-bead (Pierce and Warriner, Chester, UK), washed and dried, was incubated with Na[^125^I] (1 mCi per 100 μg protein) for 5 min in 100 μl PBS. LMF (100 μg protein) was then added and the reaction allowed to proceed for 15 min. The iodo-bead was physically removed and free Na[^125^I] was removed using a Sephadex G25 column eluted with 0.1 M NaI. The [^125^I] LMF was concentrated using a Microcon microconcentrator with a M_r_ 10 000 cut-off against PBS.

### Binding studies

CHOK1 cells transfected with the human β3-AR were lysed by sonication in 0.5 M MgCl_2_, 2 mM Tris HCl, pH 7.5 and crude membranes were pelleted by centrifugation (45 000 **g**, 15 min, 4°C). Binding studies were conducted in 400 μl 0.5 mM MgCl_2_ 50 mM Tris HCl, pH 7.5, by incubation of membranes (50 μg protein) with various concentrations of [^125^I] LMF for 60 min at 37°C. The samples were then centrifuged at 13 000 *g* for 20 min, the supernatant was removed and the radioactivity of the pellet was determined using a Packard Cobra Model 5005 Auto-gamma counter. Binding was analyzed using non-linear regression analysis (GraphPad Prism, Version 3.00 for windows, GraphPad Software (San Diego, CA, USA)).

## RESULTS

**Figure 1:**
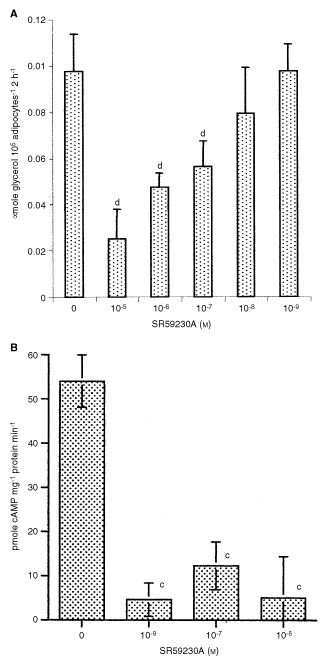
(**A**) Effect of the β3-AR antagonist SR59230A on lipolysis in murine white adipoctes; induced by LMF. Adipocytes were preincubated with the indicated concentration of SR59230A for 30 min prior to the addition of LMF (465 nM). (**B**) Effect of SR59230A on the stimulation of adenylate cyclase in isolated murine adipocyte plasma membranes by LMF. Membranes were preincubated with the indicated concentrations of SR59230A for 30 min prior to the addition of LMF (2.35 μM) and adenylate cyclase was determined as described in Materials and Methods in the presence of 0.1 μM GTP. The results are expressed as means ±s.d. and the data is representative of three separate experiments. Differences from incubation in the absence of SR59230A is indicated c, *P*<0.005 and d, *P*<0.001 as determined by Student's *t*-test.

LMF induced a direct lipolytic response in murine white adipocytes, and this effect was attenuated by low concentrations (10^−5^–10^−7^ M) of SR59230A (Figure 1A

), which has been reported to have a 10-fold selectivity for the β3-AR over the β1-AR (Nisoli *et al*, 1996). Induction of lipolysis by LMF was associated with a stimulation of adenylate cyclase in isolated adipocyte membranes in the presence of 0.1 μM GTP, and this action was almost completely inhibited by SR59230A at concentrations as low as 10^−9^ M (Figure 1B). The difference in sensitivity of intact adipocytes and plasma membranes may be related to access of SR59230A to the β3-AR. SR59230A has been shown to bind strongly to albumin (Nisoli *et al*, 1996) reducing the effective concentration available in the adipocyte assay. These results suggest that LMF stimulates lipolysis through interaction with a β3-AR.

**Figure 2:**
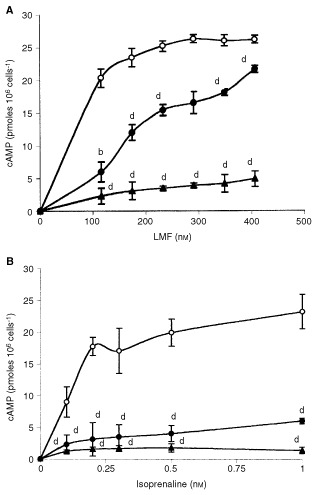
Effect of LMF (**A**) and isoprenaline (**B**) on cyclic AMP levels in CHOK1β3 cells in the absence (○) or presence of 10 μM propranolol (•) or 10 μM SR59230A (▴) Differences from controls are indicated as b, *P*<0.01 and d, *P*<0.001 as determined by ANOVA.

To investigate this possibility the effect of LMF on cyclic AMP production was determined in CHOK1 cells, which had been transfected with the human β3-AR. The data presented in Figure 2

shows that both isoprenaline and LMF stimulated cyclic AMP production, which reached a comparable maximum level of 25 pmoles per 10^6^ cells with both agents. However maximal cyclic AMP production was achieved with much lower concentrations of isoprenaline (1 nM) than LMF (250 nM), suggesting that LMF had a lower affinity for the β3-AR than isoprenaline. The increase in intracellular cyclic AMP produced by both isoprenaline and LMF in CHOK1β3 was attenuated by the non-specific β-AR antagonist propranolol (10 μM), while the effect on LMF, although significant, was less than complete. However, cyclic AMP production by both isoprenaline and LMF was almost completely attenuated by SR59230A, confirming that the action of LMF was mediated through a β3-AR.

To determine the affinity of binding of LMF to the β3-AR, LMF was radioiodinated with ^125^I and the binding to crude plasma membranes from CHOK1β3 cells was determined. The data is presented in Table 1

**Table 1:**
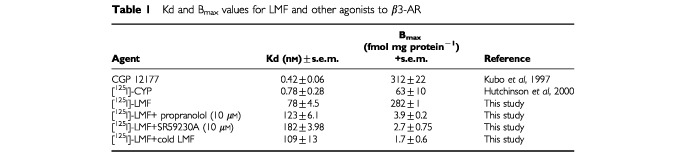
Kd and B_max_ values for LMF and other agonists to β3-AR

. Non-linear regression analysis of binding showed a high affinity binding site for LMF with a Kd value about 100-fold lower than that of CGP 12177, a partial agonist of β3-AR (Kubo *et al*, 1997) and [^125^I] iodocyanopindolol (Hutchinson *et al*, 2000), commonly used in binding studies with β3-AR. However, the B_max_ value for LMF was similar to that for other β3-AR agonists. Binding of [^125^I] LMF was significantly reduced in the presence of non-labelled LMF, the non-specific β-AR antagonist propranolol and the selective β3-AR antagonist SR59230A (Table 1). These results confirm that LMF binds to a β3-AR and stimulates adenylate cyclase.

## DISCUSSION

Resting energy expenditure (REE) has been reported to be significantly increased in weight losing patients with lung (Fredrix *et al*, 1990) and pancreatic cancer (Falconer *et al*, 1994). Hyltander *et al* (1991) found that cancer patients had an elevated REE and increased fat oxidation compared with either weight losing or weight stable controls, and that this was related to an increased heart rate. Such patients were also found to exhibit an increased cardiovascular and metabolic response to adrenaline infusion (Drott *et al*, 1987), while administration of the non-specific β-blocker propranolol was found to produce a decrease in the basal metabolic rate (BMR) (Gambardella *et al*, 1999). These results led to the hypothesis of overactivity of the sympathetic nervous system (SNS) in cancer patients.

Classical β1 and β2-AR mediate response to noradrenaline released from the SNS. In addition a third β-AR subtype has been identified (reviewed in Howe, 1993), which shares only 40–50% amino acid sequence identity with β1 and β2-AR, and is referred to as a β3-AR. These receptors mediate lipolysis in white adipose tissue in mice and rats (Arch *et al*, 1984; Arch and Wilson, 1996), and thermogenesis in BAT (Arch, 1989), and are also responsible for the unexpected negative inotropic effects of catecholamines in the heart (Gauthier *et al*, 1996). However, the evidence that β3-AR can mediate lipolysis in human adipocytes is controversial, since β3-AR mRNA is expressed at a much lower level than in rat or mouse (Langin *et al*, 1991), although lipolysis has been induced in human omental fat cells by the selective β3-AR agonist CGP 12177 (Hoffstedt *et al*, 1995), and LMF (Hirai *et al*, 1998).

We have previously shown that cachexia in both mice and humans is associated with LMF production by the tumour and excretion in the urine (Todorov *et al*, 1998), and that LMF stimulated lipolysis like a classical lipolytic hormone through increases in intracellular cyclic AMP as a result of the stimulation of adenylate cyclase (Hirai *et al*, 1997). This study shows that LMF exerts this effect through a β3-AR, although the affinity for this receptor appears to be less than seen with classical β3-AR agonists. In white adipocytes both the induction of lipolysis and the stimulation of adenylate cyclase were attenuated by the β3-AR antagonist SR59230A (Nisoli *et al*, 1996), while in CHO cells transfected with the human β3-AR LMF stimulated cyclic AMP production in a similar manner to isoprenaline, although the concentration required to produce maximal stimulation was 250-fold greater. In addition SR59230A attenuated the increase in cyclic AMP confirming the effect was mediated through a β3-AR. The effect of propranolol was less complete than with isoprenaline, suggesting that the mechanism of stimulation by LMF may be different. Previous studies (Khan and Tisdale, 1999) have shown propranolol to act as a non-compedative inhibitor of the induction of lipolysis in murine white adipocytes by LMF. This suggests that it may act at a site distal to the β3-AR and may attenuate the action of two β3 agonists to different extents. In this study we have used intact cells, since the coupling efficiency of β3-AR to adenylate cyclase is highly dependent upon the integrity of the cells (Granneman, 1995). However, it is known that the coupling efficiency of β3-AR is greater than that for β1-AR, thus offsetting the low binding affinity. Also unlike β1 and β2-AR the β3-AR has fewer potential phosphorylation sites and is resistant to agonist-induced desentitization (Granneman, 1995). The β3-AR mediated coupling of LMF to lipolysis would explain the lowered maximal response of human omental adipocytes to lipolysis when compared with murine white adipocytes (Hirai *et al*, 1998). However, the increased coupling efficiency together with the induction of UCP1 in brown adipose tissue (BAT) (Russell *et al*, 2000) would ensure maximum fat mobilization and utilization together with a net increase in energy expenditure. These results suggest that selective β3-AR antagonists may be useful in controlling energy expenditure and fat mobilization in cancer cachexia.
